# Different Effects of 2 mA and 4 mA Transcranial Direct Current Stimulation on Muscle Activity and Torque in a Maximal Isokinetic Fatigue Task

**DOI:** 10.3389/fnhum.2020.00240

**Published:** 2020-06-25

**Authors:** Craig David Workman, Alexandra C. Fietsam, Thorsten Rudroff

**Affiliations:** ^1^Department of Health and Human Physiology, College of Liberal Arts and Sciences, University of Iowa, Iowa City, IA, United States; ^2^Department of Neurology, Carver College of Medicine, University of Iowa Hospitals and Clinics, Iowa City, IA, United States

**Keywords:** tDCS, fatigue, electromyograhy (EMG), torque, motor cortex

## Abstract

Studies investigating the effects of transcranial direct current stimulation (tDCS) on fatigue and muscle activity have elicited measurable improvements using stimulation intensities ≤2 mA and submaximal effort tasks. The purpose of this study was to determine the effects of 2 mA and 4 mA anodal tDCS over the primary motor cortex (M1) on performance fatigability and electromyographic (EMG) activity of the leg muscles during a maximal isokinetic task in healthy young adults. A double-blind, randomized, sham-controlled crossover study design was applied. Twenty-seven active young adults completed four sessions, each spaced by 5–8 days. During session 1, dominance was verified with isokinetic strength testing, and subjects were familiarized with the fatigue task (FT). The FT protocol included 40 continuous maximum isokinetic contractions of the knee extensors and flexors (120°/s, concentric/concentric). During Sessions 2–4, tDCS was applied for 20 min with one of three randomly assigned intensities (sham, 2 mA or 4 mA) and the FT was repeated. The anode and cathode of the tDCS device were placed over C3 and the contralateral supraorbital area, respectively. A wireless EMG system collected muscle activity during the FT. The 2 mA tDCS condition had significantly less torque (65.9 ± 32.7 Nm) during the FT than both the sham (68.4 ± 33.9 Nm, *p* < 0.001) and 4 mA conditions (68.4 ± 33.9 Nm, *p* = 0.001). Furthermore, the 2 mA condition (33.8 ± 11.7%) had significantly less EMG activity during the FT than both the sham (39.7 ± 10.6%, *p* < 0.001) and 4 mA conditions (40.5 ± 13.4%, *p* = 0.001). Contrary to previous submaximal isometric fatigue investigations, the 2 mA tDCS condition significantly reduced torque production and EMG activity of the leg extensors during a maximal isokinetic FT compared with the sham and 4 mA conditions. Also, torque production and EMG activity in the 4 mA condition were not significantly different from sham. Thus, the effects of tDCS, and the underlying mechanisms, might not be the same for different tasks and warrants more investigation.

## Introduction

Transcranial direct current stimulation (tDCS) is a non-invasive means of increasing brain excitability (Nitsche and Paulus, 2000). It has been used for several years and in many populations to improve physical and psychological outcomes (Chhatbar et al., 2017). Although many tDCS devices are capable of a range of stimulation intensities (e.g., 0 mA–4 mA), most tDCS studies have used intensities of 2 mA or less and have elicited various measurable improvements (Bikson et al., 2016). However, if and how higher intensities might expand these outcomes have not been explored (Nitsche and Bikson, 2017). Early studies examining the safety of tDCS for human subjects used careful and moderate stimulation approaches (Bikson et al., 2016). However, recent studies have shown that intensities up to 4 mA are safe, tolerable, and do not elicit any serious adverse effects (Bikson et al., 2016; Workman et al., 2019, 2020b; Khadka et al., 2020). Now that the safety and tolerability of tDCS at higher intensities is better established, work exploring the performance differences between moderate (i.e., 2 mA) and higher (i.e., 4 mA) intensities is necessary to determine if increasing intensity further enhances outcomes.

Fatigue is “the decrease in physical and/or mental performance that results from changes in central, psychological, and/or peripheral factors” (Rudroff et al., 2016) and is commonly examined in tDCS studies. Previous researchers have investigated performance fatigability, defined as “the magnitude or rate of change in a performance criterion relative to a reference value over a given time of task performance” (Rudroff et al., 2016), in healthy subjects (see Angius et al., 2018b for a review) and in people with neurological disorders (Ferrucci and Priori, 2014; Tecchio et al., 2014; Lefaucheur et al., 2017; Cancelli et al., 2018). Theoretically, increased corticospinal excitability induced by tDCS, together with alterations in motor unit recruitment strategies (Krishnan et al., 2014), could lead to improvements in performance fatigue. However, the results of such studies are conflicting. Some have reported increases in time to task failure during submaximal isometric contractions (Cogiamanian et al., 2007; Williams et al., 2013; Abdelmoula et al., 2016; Angius et al., 2016; Oki et al., 2016; Radel et al., 2017; Alix-Fages et al., 2019) or maximal cycling (Okano et al., 2015; Vitor-Costa et al., 2015; Angius et al., 2018a; Lattari et al., 2018; Alix-Fages et al., 2019) and others have reported no effects in isometric tasks (Kan et al., 2013; Muthalib et al., 2013; Flood et al., 2017) or isokinetic fatigue testing (Hameau et al., 2018). Additionally, a recent study by Giboin and Gruber (2018) showed that both anodal and cathodal tDCS at an intensity of 2 mA decreased torque output and muscle activity of the knee extensors during an intermittent maximal isometric fatigue task (FT) in young, healthy male participants. However, a comparison between tDCS studies is complicated by the lack of standardized protocols (intensity, stimulation time, electrode location) and inconsistent definitions of fatigue. Furthermore, most of the aforementioned investigations used submaximal isometric contractions at lower tDCS intensities (≤ 2 mA).

Surface electromyography (EMG) provides a means of investigating the effects of tDCS on the neural drive to the muscles. Only a few have reported increased EMG activity in conjunction with tDCS (Krishnan et al., 2014; Kamali et al., 2019), while most have reported no effects (Cogiamanian et al., 2007; Kan et al., 2013; Cattagni et al., 2019; Oki et al., 2019) or detrimental effects (Giboin and Gruber, 2018). In contrast, studies investigating cortico-muscular and intermuscular coherence found increased coherence with anodal tDCS (Power et al., 2006; Dutta and Chugh, 2011; Bao et al., 2019). Thus, the influence of tDCS on EMG is uncertain. Furthermore, the above studies involved maximal/submaximal isometric testing (usually of the upper extremity), gait, or standing postural control with intensities ≤2 mA. None have investigated: (1) the effects of a lower (2 mA) intensity tDCS on leg muscle activity during a maximal isokinetic fatigue test in young healthy adults; or (2) compared these muscle activity changes with a higher (4 mA) intensity.

Therefore, the purpose of this study was to determine the effects of 2 mA and 4 mA anodal tDCS over the primary motor cortex (M1) on performance fatigability (as defined above; Rudroff et al., 2016) and EMG activity of the leg muscles in healthy young adults. It was hypothesized that both intensities would decrease leg muscle fatigability and that the 4 mA intensity would yield greater decreases in fatigability than the 2 mA intensity. Furthermore, it was hypothesized that decreased performance fatigability would be accompanied by a modulated neural drive to the leg muscles, as indicated by surface EMG.

## Materials and Methods

### Subjects

Because the effects of 4 mA tDCS on performance fatigability of healthy subjects is unknown (Angius et al., 2018b), an *a priori* sample size calculation was not possible. Therefore, a larger sample size (e.g., *n* > 20) was recruited to help ensure sufficient statistical power to detect potential differences. Therefore, 27 active young adults (*n* = 0 failed screening; see criteria below) participated in this study (females = 16; mean ± SD, age = 24.8 ± 3.3 years, height = 169.2 ± 10.5 cm, weight = 72.1 ± 13.4 kg). The inclusion criteria were 18–30 yrs. old, right-side dominant, undertaking ≥ 30 min of moderate-intensity physical activity ≥ 3 days/week for the previous 3 months, not taking psychoactive medications, and no chronic neurological, psychiatric, or medical conditions. The exclusion criteria included pregnancy, holes or fissures in the skull, and metal objects or implanted devices in the skull (e.g., metal plate). The study was performed following the Declaration of Helsinki. The University of Iowa’s Institutional Review Board approved this study and all subjects provided written informed consent before beginning participation.

### Experimental Protocol

**Figure 1:**
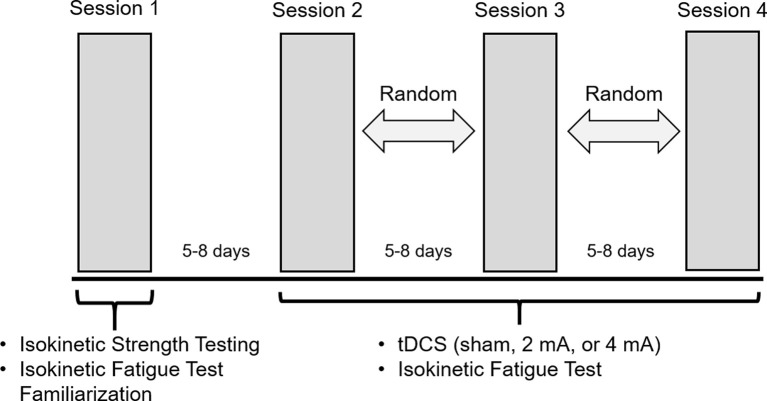
Experimental protocol. Subjects completed four sessions, with 5–8 days between each session. During Session 1, subjects performed isokinetic strength testing to verify right-side dominance and were familiarized with the isokinetic fatigue test (FT). During Sessions 2–4, transcranial direct current stimulation (tDCS) was applied with one of the three randomly assigned intensities (sham, 2 mA, 4 mA) for 20 min. Subjects started the FT at minute 15 of the tDCS application.

A double-blind, randomized, sham-controlled crossover study design was applied. Subjects completed four sessions, each spaced by 5–8 days. During Session 1, dominance was verified with isokinetic strength testing (details below). Right-side dominant subjects were exclusively recruited to avoid brain morphology differences between left- and right-dominant people (Jang et al., 2017). To familiarize each subject with the fatigue protocol used in remaining sessions and to mitigate any learning effects, the subjects also completed the isokinetic fatigue test (FT: details below) on the right leg in Session 1. During Sessions 2–4, tDCS was applied with one of three randomly assigned intensities (sham, 2 mA or 4 mA; details below) and the FT was repeated (Figure 1).

### Isokinetic Strength Test

The isokinetic testing, both strength, and fatigue were completed on a HUMAC NORM isokinetic dynamometer (CSMi, Stoughton, MA, USA). The strength test was preceded by a 15 repetition submaximal warm-up of the knee extensors and flexors (60°/s, concentric/concentric). After a short rest interval (≥30 s), the subjects performed maximal effort knee extension and flexion of the right leg (60°/s, concentric/concentric) in five sets of one repetition (Montenegro et al., 2015), with ≥30 s rest between sets. The left leg strength test was performed in the same manner as the right. The highest peak torque of the five sets was retained for dominance verification. To help ensure maximal effort, online visual feedback (i.e., a bar graph of the work performed) and verbal encouragement were provided to each subject.

### Isokinetic Fatigue Test

The FT protocol included 40 continuous maximum contractions of the knee extensors and flexors (120°/s, concentric/concentric; Saenz et al., 2010) of the right leg. Similar protocols have been used in various populations (Thorstensson and Karlsson, 1976; Lambert et al., 2001; Hameau et al., 2018; Mackey et al., 2018; Ciccone et al., 2019). In Sessions 2–4, the subjects performed the same 15 repetitions submaximal warm-up described above. Then, at the appropriate time during tDCS administration (see below), the FT was performed. Online visual feedback (i.e., a series of work bars) and verbal encouragement were again provided to encourage a maximal effort for each repetition. The peak torque achieved in each repetition was retained for analysis.

### Electromyography

**Figure 2:**
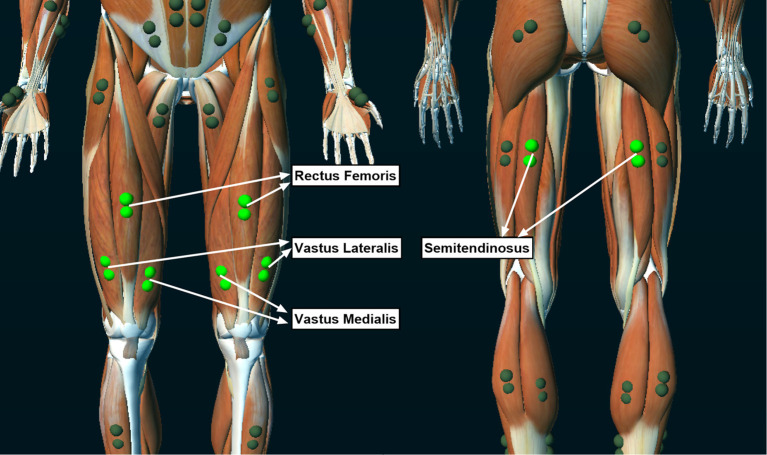
Locations of the electromyographic (EMG) electrodes that collected muscle activity during the isokinetic fatigue test.

A wireless EMG system (Ultium-EMG, Noraxon USA Inc., Scottsdale, AZ, USA) was used to collect muscle activity during strength and fatigue testing. EMG electrodes were placed and oriented over the rectus femoris, vastus medialis, vastus lateralis, and semitendinosus muscles according to the 3D Muscle Map provided by the EMG software (MR 3.14, myoMUSCLE, Noraxon USA Inc., Scottsdale, AZ, USA; Figure 2). The electrode sites were shaved and vigorously cleaned with alcohol wipes before applying the dual EMG electrodes (Noraxon USA Inc., Scottsdale, AZ, USA; 2 cm between each 1.3 cm diameter electrode) over the muscles. The wireless transmitters and electrodes were secured in place with elastic bandages. EMG data were collected at 2,000 Hz.

### tDCS Stimulation Protocol

**Figure 3:**
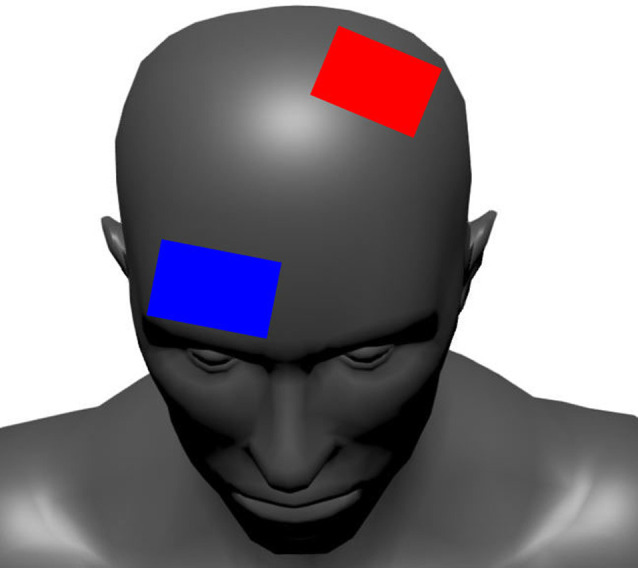
The tDCS electrode configuration. The red electrode represents the anode (positive) and the blue electrode represents the cathode (negative).

This tDCS methodology and set-up have been described elsewhere (Workman et al., 2019). Briefly, a battery-powered 1X1 tDCS device (Soterix Medical Inc., New York, NY, USA) delivered the tDCS stimulation. Two carbon electrodes were placed inside 0.9% NaCl saline soaked 5 cm × 7 cm sponges (35 cm^2^ area; EASYpad, Soterix Medical Inc., New York, NY, USA). The current density of the 2 mA and 4 mA intensities was 0.06 mA/cm^2^ and 0.11 mA/cm^2^, respectively. The anode and cathode were placed over C3 (10–20 EEG convention) and the contralateral supraorbital area (Figure 3). This anodal location was chosen to unilaterally target the dominant M1 (Jayaram and Stinear, 2009; Foerster et al., 2018). Furthermore, the electrode abutted or covered the center of the skull (Cz) and covered the leg area of M1 located in the longitudinal fissure (Foerster et al., 2018) in all subjects. The electrodes were held in place with an EASYstrap (Soterix Medical Inc., New York, NY, USA). The 2 mA and 4 mA tDCS conditions started with a 30 s ramp-up to the desired intensity, which was maintained for 20 min before a 30 s ramp-down. For sham, the device automatically administered the 30 s ramp-up to 2 mA followed immediately by a ramp-down to 0 mA. The intensity remained at 0 mA for 20 min, after which another ramp-up/ramp-down procedure was automatically administered by the device.

Before administering tDCS, the contact quality of the electrodes was checked with the device’s “PRE-STIM TICKLE” function. This function activates a 1 mA stimulation intensity for ~ 30 s and helped ensure the electrodes were adequately soaked and had firm scalp contact. In Session 2 (the first tDCS session), the location of the anode and cathode on the EASYstrap were recorded and the electrodes were positioned in the same place for Sessions 3 and 4. tDCS was administered with the subject seated in the dynamometer chair. The subjects started the FT at minute 15 of the 20 min stimulation time. Thus, tDCS was delivered both before and during the FT.

To assess the tolerability of the stimulation, the subjects reported any sensations felt during stimulation (e.g., burning, itching, tingling; Aparício et al., 2016), and rated the severity of those sensations on a 10-point Likert-type scale (1 = “barely perceptible,” 10 = “most I could stand”). To assess the integrity of the stimulation blinding, the subjects also guessed which intensity they experienced (sham, 2 mA, 4 mA) in a given session, but feedback about the accuracy of their guesses was not provided until the last session was completed. The same test administrator controlled the tDCS device for all subjects in all conditions. Both the remaining testers and the subjects were blind to the stimulation parameters.

### Data Analysis

The EMG signals from each muscle were bandpass filtered (3.5 Hz–350 Hz; Radel et al., 2017), rectified, smoothed (root-mean-square, 50 ms window), and normalized to the highest EMG peak obtained during strength testing. The average of the normalized EMG activity during each repetition at each muscle was calculated during the respective knee extensor and flexor active phases. Furthermore, because torque production during knee extension represented the contribution of all of the knee extensor muscles, the muscle activity of the knee extensors (rectus femoris, vastus medialis, and vastus lateralis) was averaged to represent the composite activity of this muscle group.

Also, it was observed that several subjects did not achieve maximum torques until the third repetition in any condition. Thus, the first two repetitions of all FTs were removed from the analysis and subsequent calculations were performed using the remaining 38 repetitions. To simplify the statistical analysis, which aimed to assess the change in torque production and EMG activity throughout the FT, the 38 repetitions were grouped into eight windows. The first seven windows represented five sequential, non-overlapping repetitions (e.g., window 1 = reps 3–7; window 2 = reps 8–12, etc.), and the last window contained the final three repetitions. EMG data were analyzed using MyoMuscle (MR3 Version 3, Noraxon USA Inc., Scottsdale, Arizona) and torque data were analyzed with MATLAB 2019a (The MathWorks, Natick, MA, USA).

### Statistical Analysis

Strength and performance differences between knee extensors and flexors are well-established (Gür et al., 1999; Coombs and Garbutt, 2002). Therefore, to help avoid Type I errors and exaggerating significance correction (below), a significant performance difference between these muscle groups was assumed and was not compared. Accordingly, a stimulation condition (sham vs. 2 mA, vs. 4 mA) by time window (1 vs. 2 vs. 3 vs. 4 vs. 5 vs. 6 vs. 7 vs. 8) repeated-measures ANOVA of the torque and average EMG was performed for the right knee extensor and knee flexor muscle groups. Paired *post hoc* analyses (*t*-tests) and effect sizes (Cohen’s *d*) were performed on any significant main or interaction effects. Significance was accepted at *p* ≤ 0.05, after a Bonferroni correction. Greenhouse–Geisser corrections were planned in cases where the repeated-measures ANOVA sphericity assumption was violated. Statistical analyses were performed using GraphPad Prism 8.1.2 for Windows (GraphPad Software, San Diego, CA, USA).

## Results

**Figure 4:**
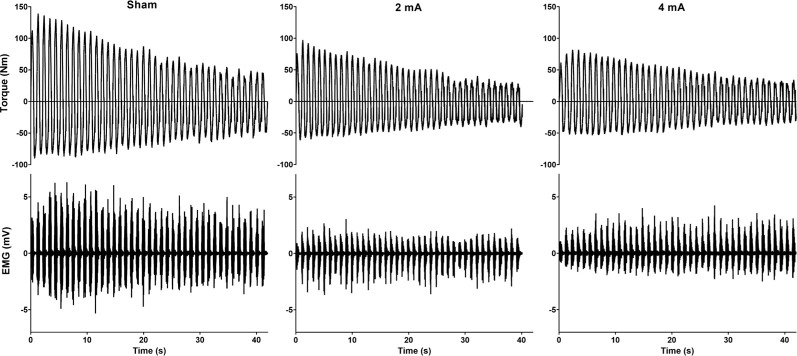
Example torque and EMG signals for the sham, 2 mA, and 4 mA tDCS conditions from a representative subject. EMG, electromyography.

All subjects completed all of the study conditions and all analysis assumptions were met. Data are reported as mean ± SD in text and mean ± SEM in the figures. Figure 4 displays example torque and EMG signals for the sham, 2 mA, and 4 mA tDCS conditions from a representative subject.

**Figure 5:**
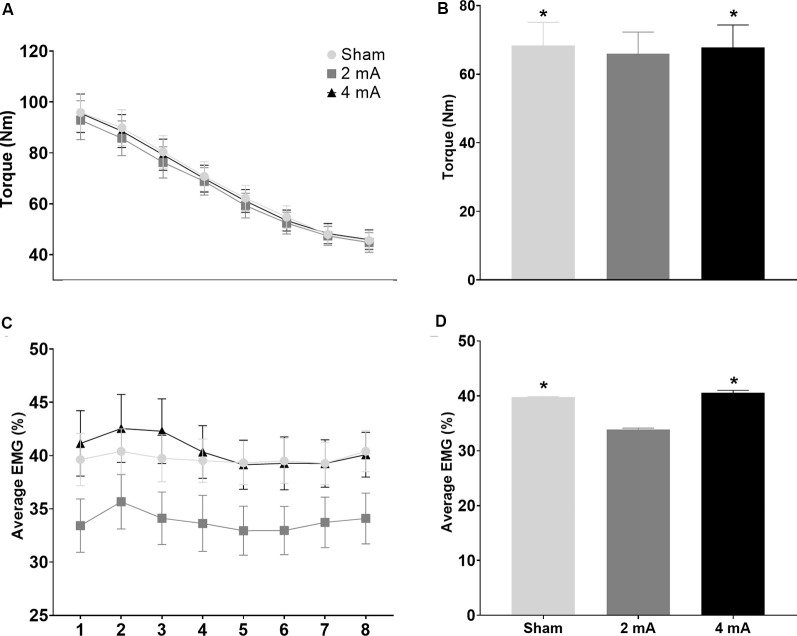
Significant main effects in torque production and EMG activity of the right knee extensors during the isokinetic fatigue test. Data are mean ± SEM. Panels **(A,C)** respectively show changes in torque production and muscle activity (average EMG %) over the eight time-windows of the isokinetic fatigue test, stratified by tDCS condition (sham, 2 mA, 4 mA). For **(A)**, torque decreased over time and each time window was significantly different from the others (e.g., 1 vs. 2, 1 vs. 3 … 7 vs. 8; significance not indicated on the figure). Panels **(B,D)** respectively show comparisons of the average torque production and muscle activity (average EMG %) stratified by tDCS condition (sham, 2 mA, 4 mA). In Panels **(B,D)**, *indicates significantly different from the 2 mA tDCS condition. EMG, electromyography; tDCS, transcranial direct current stimulation.

**Figure 6:**
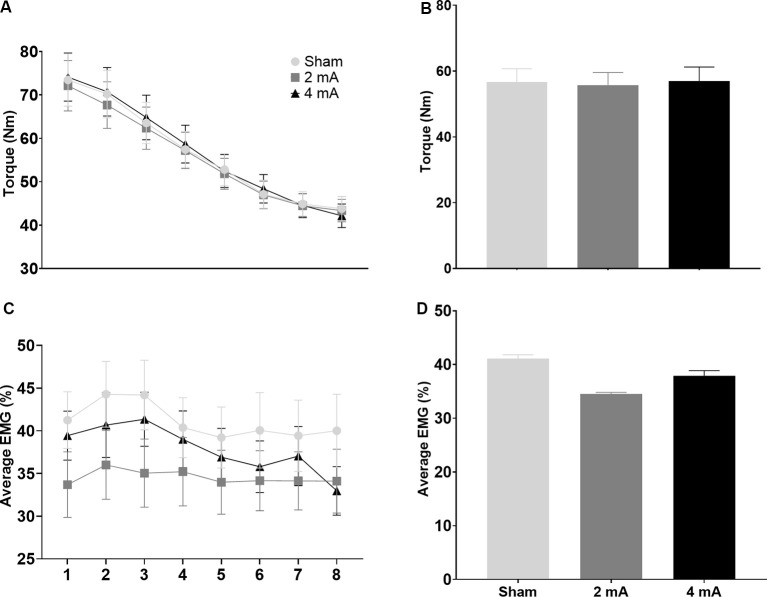
Significant main effects in torque production and EMG activity of the right knee flexors during the isokinetic fatigue test. Data are mean ± SEM. Panels **(A,C)** respectively show torque production and muscle activity (average EMG %) over the eight time-windows of the isokinetic fatigue test, stratified by tDCS condition (sham, 2 mA, 4 mA). For **(A)**, torque decreased over time and each time window was significantly different from the others (e.g., 1 vs. 2, 1 vs. 3 … 7 vs. 8; significance not indicated on the figure). Panels **(B,D)** respectively show torque production and muscle activity (average EMG %) stratified by tDCS condition (sham, 2 mA, 4 mA). EMG, electromyography; tDCS, transcranial direct current stimulation.

Figures 5A,C show the change in torque production and EMG activity of the right knee extensors over the eight time-windows of the FT for the three tDCS conditions (sham, 2 mA, 4 mA). Figures 5B,D show comparisons of the average torque production and EMG activity of the right knee extensors for the sham, 2 mA, and 4 mA tDCS conditions. Figures 6A–D show the same data for the right knee flexors. The repeated-measures ANOVA indicated significant stimulation condition and time window main effects for the torque of the right knee extensors (*F*_(7,364)_ = 3.115, *p* = 0.05 and *F*_(7,364)_ = 93.54, *p* < 0.001, respectively), but not an interaction effect (*F*_(7,364)_ = 0.789, *p* = 0.54). Pairwise testing for stimulation condition indicated that the 2 mA condition (65.9 ± 32.7 Nm) had significantly less torque during the FT than both the sham (68.4 ± 33.9 Nm, *p* < 0.001, *d* = 0.07) and 4 mA conditions (68.4 ± 33.9 Nm, *p* = 0.001, *d* = 0.06; Figure 5B). The pairwise testing for the time window effect indicated significant differences between all windows (e.g., 1 vs. 2, 1 vs. 3 … 7 vs. 8), with torque significantly decreasing over time (all *p* < 0.001, *d* range = 3.5–39.9; Figure 5A). There was a significant time window main effect for the torque of the right knee flexors (*F*_(7,364)_ = 61.43, *p* < 0.001), but not a stimulation condition or interaction effect (*F*_(7,364)_ = 0.924, *p* = 0.40 and *F*_(7,364)_ = 1.726, *p* = 0.11, respectively). Similar to the knee extensors, pairwise testing of the knee flexors revealed significant decreases in torque production with time (i.e., between all-time windows; all *p* < 0.02, *d* range = 0.02–0.65; Figure 6A).

For the average EMG, there was only a significant stimulation condition main effect for the right knee extensors (*p* = 0.02), but not a time window or interaction effect (*p* = 0.22 and *p* = 0.49, respectively). The paired *t*-tests indicated that the 2 mA condition (33.8 ± 11.7%) had significantly less EMG activity during the FT than both the sham (39.7 ± 10.6%, *p* < 0.001, *d* = 0.53) and 4 mA conditions (40.5 ± 13.4%, *p* = 0.001, *d* = 0.53; Figure 5D). There were no significant stimulation condition, time window, or interaction effects for the right knee flexors (*p* = 0.18, *p* = 0.10, and *p* = 0.74, respectively; Figure 6A).

The most common sensations reported in the three tDCS conditions were tingling (sham: 1.5 ± 1.0, *n* = 13; 2 mA: 2.2 ± 1.0, *n* = 16; 4 mA: 2.8 ± 1.6, *n* = 11), burning (sham: 3.1 ± 1.5, *n* = 10; 2 mA: 2.5 ± 2.1, *n* = 13; 4 mA: 4.6 ± 1.7, *n* = 18), and itching (sham: 2.3 ± 1.3, *n* = 8; 2 mA: 3.8 ± 1.9, *n* = 15; 4 mA: 3.5 ± 2.0, *n* = 12) which were all considered mild. There were also a few moderate—severe sensations reported in the 2 mA condition (spike: 6.0 ± 0.0, *n* = 1) and in the 4 mA condition (headache: 7.0 ± 1.4, *n* = 2; pressure: 7.0 ± 0.0, *n* = 1). For stimulation blinding, 65.0%, 51.9%, and 37.0% of subjects correctly guessed the sham, 2 mA, and 4 mA conditions, respectively.

## Discussion

It was hypothesized that anodal tDCS would decrease leg muscle fatigability, which would be accompanied by a modulated neural drive (EMG) to these same muscles. The findings of this study do not support this hypothesis. The main and novel results of this study are: (1) 2 mA tDCS reduced torque production of the knee extensors during a maximal isokinetic FT, accompanied by reduced EMG activity; and (2) 4 mA tDCS did not affect torque production and EMG activity compared to sham.

The present study applied sham, 2 mA, and 4 mA tDCS for 15 min before performing the FT during the remaining 5 min of stimulation (20 min total). Previous studies have indicated that 15 min of tDCS is sufficient to induce after-effects (Nitsche and Paulus, 2000). However, the stimulation in this study resulted in detrimental effects on the FT performance. One potential explanation is that the expected tDCS effects were blunted by the performance of the maximal FT. Thus, submaximal isometric contractions may be more sensitive to tDCS after-effects than maximal tasks. Furthermore, the initial maximal contractions may have influenced N-methyl-D-aspartate (NMDA) receptor efficacy (Nitsche et al., 2003), and resulted in depressed stimulation after-effects. Also, because tDCS was also applied during the FT, it may be that the direct effects of tDCS, i.e., changes in membrane polarization and neurotransmitter release (Márquez-Ruiz et al., 2012), were dampened, and thus were unable improve maximal force production, especially at the 2 mA intensity.

Only a few tDCS studies have investigated the effects of tDCS on maximal contractions, with contrasting findings. Sales et al. (2016) investigated the effects of 2 mA tDCS applied over the left temporal lobe before an isokinetic muscle performance test consisting of two sets of five repetitions, one at 60°/s and another at 180°/s. The performance of both tested velocities showed significant improvements in the total work performed with tDCS compared to sham. Giboin and Gruber (2018), on the other hand, found detrimental effects of both anodal and cathodal tDCS at 2 mA. In that study, the stimulation was applied before or during an intermittent maximal isometric FT. Both anodal and cathodal tDCS reduced MVC amplitude (aMVC) when tDCS was applied during the task, and only anodal tDCS reduced aMVC when it was applied 10 min before the task. These reductions in aMVC were accompanied by reductions in EMG of the vastus lateralis. We concluded that tDCS might not be an adequate performance enhancement tool for all tasks or types of effort (e.g., maximal vs. submaximal). The effects of 2 mA tDCS in the current study are as per Giboin and Gruber (2018). The conflicting performance fatigability effects in previous submaximal isometric fatiguing studies (Cogiamanian et al., 2007; Williams et al., 2013; Abdelmoula et al., 2016; Angius et al., 2016; Oki et al., 2016; Radel et al., 2017; Lattari et al., 2018) and the maximal MVCs in the present and Giboin and Gruber’s (2018) studies might be explained by different fatigue mechanisms on which tDCS may act in different FTs. As quoted above, fatigue is defined as “the decrease in physical and/or mental performance that results from changes in central, psychological, and/or peripheral factors” (Rudroff et al., 2016). Thus, given that the outcome of a tDCS intervention depends on several factors, such as intensity and timing of tDCS, the task being performed, the environmental conditions in which it is performed, and the physical and mental capacity of the individual subject, it seems that task specificity plays an important role in tDCS applications and outcomes.

The greater reduction of MVC torques induced by 2 mA tDCS might be explained by increased agonist/antagonist co-contraction. However, increased knee flexor activation was not observed in this study. Also, surface EMG has known limitations of (e.g., amplitude cancellation, cross-talk; Farina et al., 2004, 2014) and may not adequately reflect changes in the neural drive to the muscles (Del Vecchio et al., 2017). Thus, the evaluated EMG parameters may not be sufficiently sensitive to detect potentially subtle changes in the central recruitment of spinal motor neurons, especially at higher intensities (e.g., 4 mA). Additionally, the effect of anodal tDCS on torque production may not arise from a postsynaptic effect on cortico-motor projections but could be related to a presynaptic effect on the motor cortex interneuronal network. Therefore, additional measures, such as voluntary activation (VA), potentiated twitch at rest, and motor evoked potentials might provide further insights (Pageaux et al., 2015).

The decline in torque production during prolonged effort is thought to be related to the “upstream” failure of motor cortical neurons (Gandevia et al., 1996; Gandevia, 2001; Taylor et al., 2006), which might be influenced by tDCS. Additionally, because there is widespread polarization of the cortex from tDCS (Baudewig et al., 2001; Lang et al., 2005), possible concomitant effects involving cortical areas adjacent to the anode cannot be overlooked. For example, a study that used functional magnetic resonance imaging (fMRI) during a sustained maximal contraction showed an initial increase in brain activity from the beginning to the middle of the task, followed by a significant reduction from the middle to the end of the task (Liu et al., 2002). This pattern of changing activity was found not only in the primary sensory and motor areas but also in the secondary and association cortices. Therefore, the performance effects of tDCS might be modulated by motor areas outside of M1 (e.g., supplementary motor area).

The lack of difference between the sham and 4 mA conditions indicates no effect of higher intensity stimulation on performance and represents an interesting finding. One explanation for this similarity may be related to the tDCS-induced modulation of the inhibitory feedback systems, which limit motor cortical output to “protect” the motor system from overload (Cogiamanian et al., 2007; Sales et al., 2016). Maximal performances also require an optimal interaction between motor cortex impulses and sensory cortex processing (Proske and Allen, 2019). Both performance fatigability and perception of fatigue may result in sub-optimal motor commands, and the tDCS stimulation may have contributed to these effects. Similarly, another potential explanation for the different torque productions and EMG activities between the stimulation conditions could be that tDCS differently affected the motivation of individual subjects to achieve maximal effort for each contraction, *via* modulation of frontal cortex activity (Schmidt et al., 2009; Blakemore et al., 2017). In this regard, stimulation-related discomfort induced by tDCS during the FT might have negatively affected the subjects’ concentration on producing and maintaining maximal efforts during the task. However, strong verbal encouragement was given to each subject to reduce these effects as much as possible.

### Limitations and Future Studies

There are a few limitations of note for this study. Surface EMG amplitude changes may not exactly reflect changes in the neural drive (Del Vecchio et al., 2017), which would make it difficult to quantify the effects of tDCS on muscle activation. VA, which requires peripheral motor unit stimulation and intentional investigation, might be an appropriate alternative measure to detect the effects of tDCS on muscle activation during FTs (Pageaux et al., 2015; Giboin et al., 2018). VA estimation represents “the drive by the motor neurons to the muscle and how it translates to force” (Taylor, 2009). Consequently, the reduction of VA during or after a FT reflects the incidence of central fatigue. Another limitation is that transcranial magnetic stimulation (TMS) equipment that measures cortical excitability was not available for this study. TMS and electromyography (EMG) together might better determine how purported changes in cortical excitability from tDCS are associated with the physiological effects (i.e., muscle activity) of motor performance. Also, assessing corticospinal excitability during or after the FT could have provided more insights into the mechanisms underpinning the observed behavioral changes. However, it must be acknowledged that the changes induced by anodal tDCS on knee extension FTs are not necessarily accompanied by detectable TMS corticospinal excitability changes (Abdelmoula et al., 2016; Angius et al., 2016). Also, a recent review concluded that only the amplitude of TMS motor evoked potentials was altered with tDCS, while other TMS measures (e.g., cortical silent period, short interval cortical inhibition) were not (Horvath et al., 2015). Taken together, these two concepts suggest that TMS might not completely capture tDCS-induced physiological changes. Additionally, approximately 50% of subjects might be classified as tDCS “non-responders” (Wiethoff et al., 2014) and clear criteria for organizing potential subjects into responders and non-responders is lacking. Thus, the inability to group subjects may explain some of the variability of the present results and may have masked the effect of tDCS on muscle activity. Additionally, the applicability of single-joint testing to functional activities may also be questionable (Kollock et al., 2015) and suggests discretion in interpreting these results to real-world, multi-joint activities. Last, subjects that experience repeated sessions of tDCS have a higher probability of compromising blinding integrity (Wallace et al., 2016) and the subjects of this study may not have been successfully blinded. However, the torque and EMG data of this study do not indicate a systematic effect of a potential lack of blinding. One solution to maintain blinding integrity would be to increase the intensity of the sham condition to match, or slightly exceed, the highest intensity applied. The theoretically stronger sensations experienced in such a sham condition might be interpreted as real stimulation by more subjects and bolster blinding integrity.

Because higher intensity (>2 mA) stimulation is still novel, future work should continue to explore the effects of tDCS at higher intensities and determine the nature of the stimulation intensity dose-response. Understanding this dose-response is particularly important because a recent review concluded that the evidence of increasing tDCS intensity to enhance outcomes was inconclusive (Esmaeilpour et al., 2018). There are also indications that increasing stimulation time and/or intensity (up to 2 mA) may shift the intended anodal tDCS effects from excitation to inhibition (Batsikadze et al., 2013; Monte-Silva et al., 2013). Another study also suggested that higher intensities (4–6 mA) might be required to get enough current through the scalp, subcutaneous tissues, and skull to affect cortical excitability (Vöröslakos et al., 2018). Also, functional neuroimaging should be included in future studies to elucidate the effects of different tDCS intensities on brain activity. Future investigations should also include clinical populations with reduced cortical activity/excitability that might experience greater benefits from higher intensity tDCS (e.g., multiple sclerosis, stroke). Indeed, a preliminary 4 mA tDCS investigation in Parkinson’s disease indicated promising effects (Workman et al., 2020a). This study also adds to the growing evidence that the performance effects of tDCS are highly variable. Thus, identification of responders and non-responders to different tDCS intensities is critical, and results similar to the present study should be replicated or refuted in larger trials before tDCS can be considered an effective ergogenic aid. Lastly, the optimal timing of high-intensity tDCS stimulation (e.g., during, before) is a key component to improving tDCS applicability and efficacy and should be systematically investigated.

## Conclusion

Compared with the sham and 4 mA tDCS conditions, 2 mA of tDCS resulted in significantly reduced torque production and EMG activity of the leg extensors during a maximal isokinetic FT. Also, torque production and EMG activity in the 4 mA condition were not significantly different from sham. These results are contrary to previous submaximal and isometric studies. Thus, the effects of tDCS, and the underlying mechanisms, might be task-specific (i.e., different for maximal vs. submaximal or isometric vs. isokinetic) and warrants more investigation. Future studies should continue to explore the effects of tDCS at higher intensities (>2 mA), particularly in clinical populations, to determine the utility of increasing stimulation intensity.

## Data Availability Statement

The raw data supporting the conclusions of this article will be made available by the authors, without undue reservation, to any qualified researcher.

## Ethics Statement

The studies involving human participants were reviewed and approved by Institutional Review Board (IRB) University of Iowa. The patients/participants provided their written informed consent to participate in this study.

## Author Contributions

CW and TR contributed to: (1) conception and design of the experiments; (2) collection, analysis, and interpretation of data; and (3) drafting the article and revising it critically for important intellectual content. AF contributed to: (1) analysis and interpretation of data; and (2) preparation of figures and tables.

## Conflict of Interest

The authors declare that the research was conducted in the absence of any commercial or financial relationships that could be construed as a potential conflict of interest.
